# Molecular epidemiology and genomic dynamics of *Pseudomonas aeruginosa* isolates causing relapse infections

**DOI:** 10.1128/spectrum.05312-22

**Published:** 2023-09-28

**Authors:** Cong Shen, Jinxiang Zeng, Dexiang Zheng, Yinglun Xiao, Jieying Pu, Li Luo, Hongyun Zhou, Yimei Cai, Liling Zhang, Meina Wu, Xuan Zhang, Guangyuan Deng, Song Li, Qiwei Li, Jianming Zeng, Zhaohui Sun, Bin Huang, Cha Chen

**Affiliations:** 1 The Second Clinical Medical College, Guangzhou University of Chinese Medicine, Guangzhou, China; 2 Department of Clinical Laboratory, The Second Affiliated Hospital of Guangzhou University of Chinese Medicine, Guangdong Provincial Hospital of Traditional Chinese Medicine, Guangzhou, China; 3 Department of Laboratory Medicine, General Hospital of Southern Theater Command, Guangzhou, China; 4 Department of Clinical Laboratory, The First Affiliated Hospital of Sun Yat-sen University, Guangzhou, China; Children's National Hospital, George Washington University, Washington, DC, USA

**Keywords:** *Pseudomonas aeruginosa*, relapse infection, prevalence, molecular epidemiology, whole-genome sequencing

## Abstract

*Pseudomonas aeruginosa* (*P. aeruginosa*) is one of the leading causes of chronic infections, including reinfection, relapse, and persistent infection, especially in cystic fibrosis patients. Relapse *P. aeruginosa* infections are more harmful because of repeated hospitalization and undertreatment of antimicrobials. However, relapse *P. aeruginosa* infection in China remains largely unknown. Herein, we performed a 3-year retrospective study from 2019 to 2022 in a tertiary hospital, which included 442 *P*. *aeruginosa* isolates from 196 patients. Relapse infection was identified by screening clinical records and whole-genome sequencing (WGS). We found that 31.6% (62/196) of patients had relapsed infections. The relapse incidence of carbapenem-resistant *P. aeruginosa* infection (51.4%) is significantly higher than that of carbapenem-susceptible *P. aeruginosa* infection (20.2%, *P* < 0.0001). These isolates were assigned to 50 distinct sequence types and sporadically distributed in phylogeny, indicating that relapsed infections were not caused by certain lineages. Fast adaptation and evolution of *P. aeruginosa* isolates were reflected by dynamic changes of antimicrobial resistance, gene loss and acquisition, and single-nucleotide polymorphisms during relapse episodes. Remarkably, a convergent non-synonymous mutation that occurs in a pyochelin-associated virulence gene *fptA* (T1056C, M252T) could be a considerable target for the diagnosis and treatment of relapse *P. aeruginosa* infection. These findings suggest that integrated utilization of WGS and medical records provides opportunities for improved diagnostics of relapsed infections. Continued surveillance of the genomic dynamics of relapse *P. aeruginosa* infection will generate further knowledge for optimizing treatment and prevention in the future.

**IMPORTANCE:**

*Pseudomonas aeruginosa* is a predominant pathogen that causes various chronic infections. Relapse infections promote the adaptation and evolution of antimicrobial resistance and virulence of *P. aeruginosa*, which obscure evolutionary trends and complicate infection management. We observed a high incidence of relapse *P. aeruginosa* infection in this study. Whole-genome sequencing (WGS) revealed that relapse infections were not caused by certain lineages of *P. aeruginosa* isolates. Genomic dynamics of relapse *P. aeruginosa* among early and later stages reflected a plasticity scattered through the entire genome and fast adaptation and genomic evolution in different ways. Remarkably, a convergent evolution was found in a significant virulence gene fptA, which could be a considerable target for diagnosis and treatment. Taken together, our findings highlight the importance of longitudinal surveillance of relapse *P. aeruginosa* infection in China since cystic fibrosis is rare in Chinese. Integrated utilization of WGS and medical records provides opportunities for improved diagnostics of relapse infections.

## INTRODUCTION

*Pseudomonas aeruginosa* (*P. aeruginosa*) is a non-fermentative and aerobic Gram-negative bacillus that is one of the leading causes of severe healthcare-associated infections, such as nosocomial pneumonia, urinary tract infections, and bloodstream infections, which results in high mortality rates and brings huge medical burdens to human society (1, 2). Carbapenems are the most effective antimicrobial agents against *P. aeruginosa* infections. However, the emergence and global dissemination of carbapenem-resistant *P. aeruginosa* (CRPA) threatened the effectiveness of antimicrobial therapy, which is identified as a global public health crisis (1, 3).

A relatively large genome (5.5–7 Mb) of *P. aeruginosa* encodes a wide array of virulence factors, regulatory genes, and catabolic functions, which provides the pathogenicity and adaptability to establish acute and chronic infections and against the different stressors in the environment with the facility to tailor its response (3, 4). Its tremendous ability to adapt greatly facilitates its capacity to cause chronic infections and became a dominant pathogen that causes persistent and chronic lung infections, which contributes to morbidity and mortality in patients with cystic fibrosis (CF) and burn injury particularly (3, 5).

Chronic infections could divide into three types, including (i) persistent infection that occurs when the bacteria causing the infection are not completely eliminated by treatment and continuously reproduce and induce inflammation in the body; (ii) relapse infection that occurs when a patient’s symptoms improve and the bacteria were not detected by the clinical laboratory after treatment but then return later infected by the same bacterial clone, often because the initial treatment did not completely eradicate the infection; and (iii) reinfection that occurs when a person was invaded by new clones (6 – 8). During chronic infections, *P. aeruginosa* isolates are facing long-term host-derived stressors and antimicrobial treatment, which are significant selective pressures for evolution and adaptation (8 – 11). Remarkably, relapse infections are more harmful since the patients could be erroneously identified as being cured which obscure evolutionary trends and complicate infection management, leading to the repeated hospitalization and undertreatment of antimicrobials (12). Previous studies have confirmed that CF patients are susceptible to establish *P. aeruginosa* infection which accounts for 95% of deaths in CF patients (13, 14). Notably, *P. aeruginosa* isolates from CF patients evolved in the phenotype and genotype which confer a fitness advantage or antimicrobial resistance, establishing persistent colonization and infection (5, 15). However, CF is the most common autosomal recessive genetic disorder among Caucasians, whereas it rarely happens in Chinese (16). Other than CF, chronic obstructive pulmonary disease (COPD), bronchiectasis, pulmonary tuberculosis, lung cancer, and interstitial lung diseases are lung-associated risk factors causing repeated lung infections (12 – 15). However, the characterizations of relapse *P. aeruginosa* infections in China are largely unknown.

The aim of this study was to investigate the incidence of relapse *P. aeruginosa* infections and reveal the genomic dynamics of these relapse *P. aeruginosa* isolates. We performed a 3-year retrospective study that included 196 subjects who were diagnosed with *P. aeruginosa* infections at the Guangzhou Provincial Hospital of Chinese Medicine from September 2019 to December 2022. The *P. aeruginosa* isolates were purified and subjected to whole-genome sequencing. The population structure, multilocus sequence typing (MLST), antimicrobial resistance genes (ARGs), and virulence factors were used to demonstrate the molecular epidemiology of relapse *P. aeruginosa* isolates. Additionally, we determined the pairwise single nucleotide polymorphism (SNP) distance and evolution of isolates from early and later stages of relapse infections with the purpose of providing information on adaptive processes that could identify genetic markers and targets of relapse *P. aeruginosa* infections.

## MATERIALS AND METHODS

### Subject inclusion and exclusion criteria

This study was approved by the Guangdong Provincial Hospital of Chinese Medicine, Guangzhou, China. The clinical isolates were routinely collected and whole-genome sequenced in the hospital. To identify the subjects with *P. aeruginosa* infections, we screened the clinical records of subjects from 1 September 2019 to 31 December 2022. The subjects were included if clinical samples were evidenced by a positive *P. aeruginosa* culture, and the patients had typical clinical infectious symptoms and abnormal laboratory markers of infections (Table S1). The patients were excluded if the clinical samples were positive for the culture of *P. aeruginosa* but had no infectious symptoms, which were regarded as colonization. The source of infection was determined as pneumonia, urinary tract infection, surgical site infection, intra-abdominal infection, catheter-related infection, or bacteremia as defined by the Centers for Disease Control and Prevention (17).

### Identification of relapse *P. aeruginosa* infections

Recurrence infection was defined as the return of *P. aeruginosa* infections after documentation of negative cultures of clinical samples or clinical improvement after completing a course of antimicrobial therapy, according to previous studies (8, 18, 19). Therefore, all patients with recurrent episodes (≥2 episodes that occurred ≥30 days apart with clinical sample culture results negative for *P. aeruginosa* in the interim) were identified. Their medical records were reviewed to verify clearance (resolution of all clinical signs of infection) between recurrences and to determine the clinical characteristics of the episodes. Recurrence was further subclassified as reinfection or relapse infection: if genomes of *P. aeruginosa* isolates from the patient were closely related to and distributed into the same lineage of the phylogeny, the recurrence was considered as a relapse infection. Conversely, if the genomic contents are greatly different and the isolates were sporadically distributed on phylogeny, the recurrent episode was considered to be reinfection.

### Isolation of *P. aeruginosa* strains

Clinical samples and cultured isolates were collected as part of clinical management and hospital surveillance in the Guangdong Provincial Hospital of Chinese Medicine. Clinical samples (urine, blood, sputum, and wound samples) from patients with infections were plated on Columbia Blood Agar (CBA) with 5% sheep blood (Luqiao, Beijing, China), except blood samples that were injected into blood culture bottles (BACT/ALERT FN/FA Plus) processing in the BacT/ALERT 3D System (bioMerieux Inc.). After the culture at 37°C in 24 hours or positive alarm of blood culture, if *P. aeruginosa* is identified as the most abundant species in clinical samples, three *P. aeruginosa* colonies were selected and stored. DNA was extracted using the boiling method. *P. aeruginosa* isolates were cultured by adding 3 mL of nutrient broth and incubating for 18–24 hours at 37°C. Subsequently, total DNA was obtained from the supernatant by boiling 1 mL of the broth at 100°C for 10–15 minutes. Species identification was first confirmed by MALDI-TOF MS (BrukerDaltonik GmbH, Bremen, Germany). Where the species could not be reliably assigned by MALDI-TOF, 16S rDNA sequencing was applied. After these processes, we randomly choose one representative *P. aeruginosa* isolate for the subsequent analysis including MIC testing and whole-genome sequencing.

### Antimicrobial susceptibility testing

The antimicrobial susceptibilities of the *P. aeruginosa* isolates were determined by using the Vitek 2 Compact system (bioMérieux, France) according to the manufacturer’s instructions. The minimum inhibitory concentrations (MICs) of cefepime, ceftazidime, imipenem, meropenem, ticarcillin-clavulanate, levofloxacin, ciprofloxacin, tobramycin, and amikacin were determined, and the results were interpreted according to Clinical and Laboratory Standards Institute (Clinical and Laboratory Standards Institute, document M100-S29) guidelines. The MICs of colistin and polymyxin E (colistin) were determined using the broth dilution method (EUCAST breakpoints, version 9.0; CLSI M100-S29). The antibiotic susceptibilities of the *P. aeruginosa* isolates are provided in Table S2.

### Whole-genome sequencing and data processing

A total of 163 *P*. *aeruginosa* isolates from 65 patients with relapse infections (identified using MALDI-TOF MS ± 16s rDNA sequencing) were subjected to whole-genome sequencing. The Qiagen Blood and Tissue Kit (Qiagen, Hilden, Germany) was used to extract genomic DNA for the *P. aeruginosa* isolate according to the manufacturer’s instructions. DNA libraries were constructed with 350-bp paired-end fragments and sequenced using an Illumina HiSeq 2000 platform (Illumina Inc., USA). The sequencing yielded >100-fold coverage sequences (~1G) of raw paired-end (150 bp) per isolate. Adaptor, unreliable reads, and low-quality bases of raw reads were trimmed using fastp with default parameters (20). SPAdes v3.13.1 with the “--careful” parameter and automatically determined k-mer values (21, 33, 55, 77) were used to perform draft genome *de novo* assembly (21). Contigs less than 500 bp in size were excluded for further analysis. WGS-based species identification was performed using JSpeciesWS v3.2.7 (22). Isolates not confirmed as *P. aeruginosa* using WGS-based species identification, and contaminated/mixed sequences, were manually excluded from subsequent analyses.

### Prediction and annotation of open reading frames

Open reading frames were predicted using prodigal v2.6.3 and annotated by Prokka v1.13.3 (23). Antimicrobial resistance genes, plasmid replicons, and virulence-associated genes were identified using SRST2 v0.2 by mapping reads to ResFinder, PlasmidFinder, and VFDB database (24 – 27); meanwhile, the locations of these traits in a draft genome level were annotated using ABRicate 0.8.7 with “--minid 75 --mincov 60” parameters (https://github.com/tseemann/abricate). *In silico* MLST of *P. aeruginosa* was assigned using PubMLST (https://pubmlst.org/) against seven housekeeping genes (*acsA*, *aroE*, *guaA*, *mutL*, *nuoD*, *ppsA*, and *trpE*). New alleles and sequence types (STs) were uploaded on the PubMLST database.

### Pan-genome analysis and phylogenetic construction

Pan-genome analyses were performed using Roary (28), and a concatenated alignment of genes shared among ≥99% of all isolates (core genome) was extracted using Mafft v7.407 (29). Core genome SNPs (cgSNPs) were extracted from this concatenated alignment using SNP sites (30). To obtain high-quality cgSNPs, we filtered SNPs that are of low quality and frequency by vcftools v0.1.17 with “--minDP 5 --max-missing 0.95 --maf 0.05” parameters (31). The paired SNP distance among all isolates was calculated by snp-dists v0.7.

A maximum likelihood (ML) phylogeny for cgSNPs was reconstructed using RAxML v8.2.10 implementing a generalized time-reversible nucleotide substitution model with a Γ distribution (GTR + G) (32). Branch lengths and bootstrap supports for bipartitions were estimated using 1,000 bootstrap replicates. The population structure was assessed using cgSNPs with hierBAPS, which was run four times using maximum clustering sizes of 20, 40, 60, and 80 (33). Sequence clusters (SCs) identified using hierBAPS were labeled on the core genome phylogeny on iTOL (https://itol.embl.de/).

### Statistical analysis

Statistical analysis was conducted using R software v3.5.3. Statistical significance between groups was calculated using a two-sided Wilcoxon rank sum test and a Fisher’s exact test. *P* values less than 0.05 were considered as statistically significant.

## RESULTS

### Identification of patients with relapse *P. aeruginosa* infections

**Fig 1:**
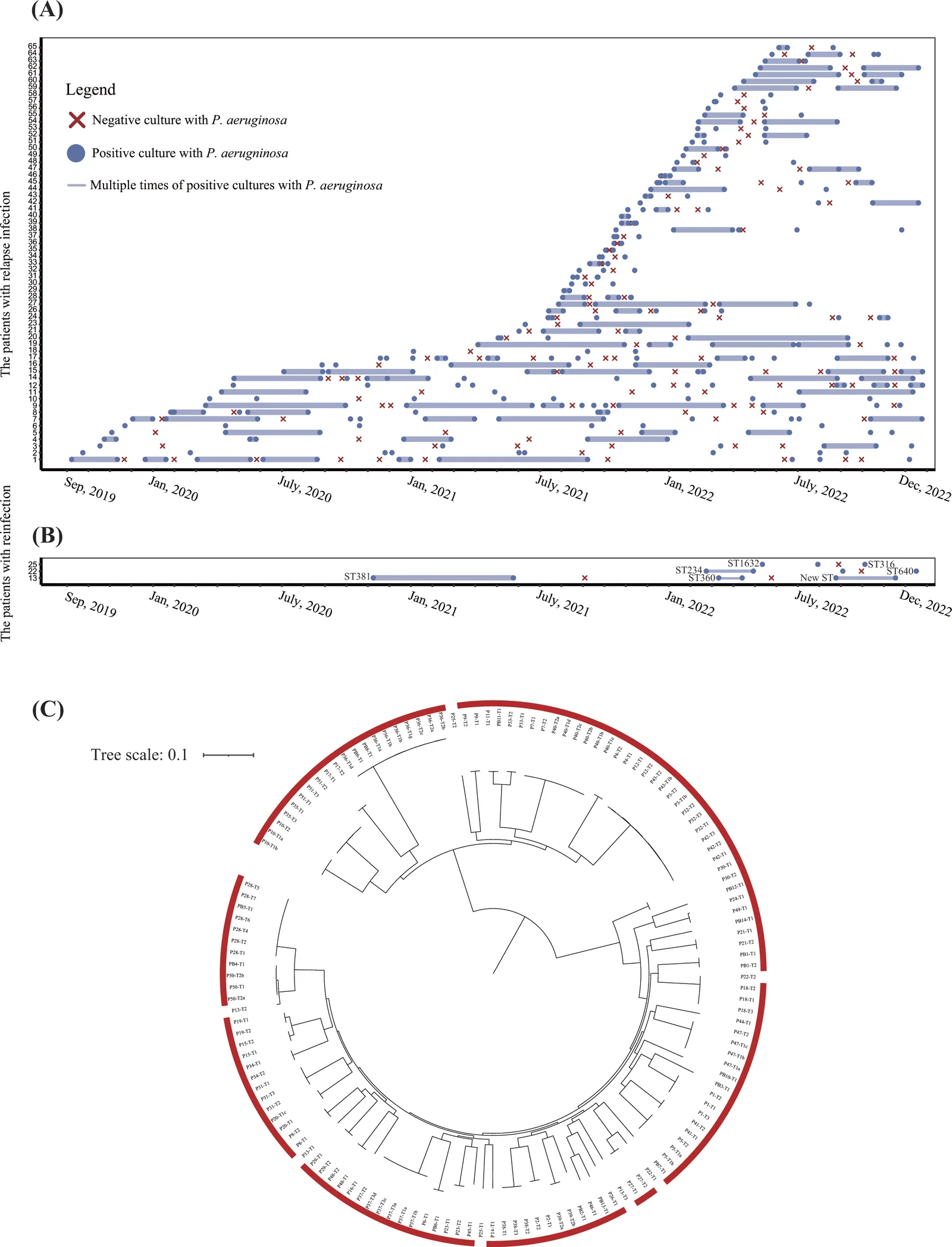
Identification of relapse infection by screening of clinical data and whole-genome sequencing. (**A**) Timelines of relapse *P. aeruginosa* infection for each patient. Data are shown for isolates of 62 patients (1–62) from 1 September 2019, to 31 December 2022. The blue dots represent the positive cultures with *P. aeruginosa* isolates. The red forks represent negative culture with *P. aeruginosa* isolates. The docs connected with solid lines represent multiple times (>2) of positive cultures with *P. aeruginosa* isolates. (**B**) Timelines of reinfection of *P. aeruginosa* for each patient. Sequence type (ST) of the isolate was annotated beside the dot. (**C**) Phylogenetic tree of 163 *P*. *aeruginosa* isolates from 65 patients with multiple times of *P. aeruginosa* infections. The tree was constructed using core genome SNPs with maximum likelihood method. The isolates labeled with red frame indicate the relapse infection, while no frame indicates reinfection.

A total of 442 non-duplicate *P. aeruginosa* isolates were identified in 196 patients at the Guangdong Provincial Hospital of Chinese Medicine from September 2019 to December 2022 (Table S1). Through screening of clinical records and inclusion criteria, we found that 33.2% (65/196) of patients had at least twice *P. aeruginosa* infections during the study period, which include relapsed infection and reinfection (Fig. 1).

We hypothesize that the relapse *P. aeruginosa* isolates should be closely related, meaning the differences in genomes should be minor and the isolates are distributed into the same lineage on phylogeny. To precisely identify the relapse *P. aeruginosa* infections, we performed WGS and phylogenetic analysis for 163 isolates from 65 patients; we identified that 156 isolates from 62 patients were distributed into the same lineage, whereas seven isolates from three patients were distributed sporadically (Fig. 1C), which indicated that 62 patients (95.4%, *n* = 65) had relapse infections and three patients (4.6%) had reinfections (Fig. 1A and B).

### Comparison of clinical characterizations among relapse and non-relapse *P. aeruginosa* isolates

Among our cohort, 66.8% (131/196) were male, with the median age being 67 years (interquartile range [IQR], 55–81 years). There are no differences in gender (male: 64.5% [40/62] vs 67.9% [91/134], *P* = 0.6388) and age distribution (≥60 years: 22.6% [14/62] vs 32.1% [43/134], *P* = 0.1782) between relapse and non-relapse infection groups (Table 1). Notably, 30.8% (20/65) of the patients were diagnosed with chronic lung diseases which were considered as risk factors in relapse *P. aeruginosa* infections (Table S1). However, only 16.9% (11//65) of patients were diagnosed with COPD, 4.6% (*n* = 3) with bronchiectasis, 7.7% (*n* = 5) with interstitial lung diseases, and 1.5% (*n* = 1) with lung cancer, and none was diagnosed with CF disease, indicating that no certain chronic lung disease could be a direct indicator.

**TABLE 1:** Clinical characteristics of 196 patients infected by *P. aeruginosa^a^*

Characteristics	Relapse infection (*N* = 62)	Non-relapse infection (*N* = 134)	*P* value
Gender			
Male	64.5% (40)	67.9% (91)	0.639
Female	35.5% (22)	32.1% (43)	
Age			
≥60 years	22.6% (14)	32.1% (43)	0.173
<60 years	77.4% (48)	67.9% (91)	
Clinical department			
Pulmonology	29.0% (18)	26.1% (35)	0.669
Rehabilitation	17.7% (11)	13.4% (18)	0.429
ICU	12.9% (8)	8.2% (11)	0.302
Neurology	12.9% (8)	7.5% (10)	0.220
Cardiology	9.7% (6)	5.2% (7)	0.392^*b*^
Neurosurgery	8.1% (5)	5.2% (7)	0.652^*b*^
Others	9.7% (6)	34.3% (46)	0.0003
Specimen source			
Sputum	64.5%(40)	54.5% (73)	0.186
Alveolar lavage fluid	21% (13)	5.2% (7)	0.001
Wound	6.5% (4)	12.7% (17)	0.189
Urine	4.8% (3)	20.9% (28)	0.004
Blood	3.2% (2)	1.5% (2)	0.799
Others	0% (0)	5.2% (7)	0.156
Clinical diagnosis			
Pneumonia	91.9% (57)	68.6% (92)	0.0003
UTI	8.1% (5)	22.4% (30)	0.0158
Wound infection	0% (0)	3.0% (4)	0.1222
BSI	0% (0)	3.0% (4)	0.1222
Pneumonia and BSI	0% (0)	1.5% (2)	0.4981
Others	0% (0)	1.5% (2)	0.4981

^*a*^ Data are presented as percentage of patients with the corresponding number in parentheses. UTI, urinary tract infection.

^*b*^ Statistical analysis with Fisher’s exact test. BSI, bloodstream infection.

Of the patients, 27.0% (53/196) were from the pulmonology department, followed by the rehabilitation department (14.8%, *n* = 29), ICU (9.7%, *n* = 19), neurology department (9.2%, *n* = 18), and other 16 departments (39.3%, *n* = 77). There is no difference in department distributions between patients with relapse and non-relapse infections (*P* > 0.05; Table 1).

The *P. aeruginosa* isolates from 57.7% (*n* = 113) of patients were collected from sputum, followed by urine (15.8%, *n* = 31), wound (10.7%, *n* = 21), and bronchoalveolar lavage fluid (10.2%, *n* = 20). The *P. aeruginosa* isolates (*n* = 6) were found in both sputum and blood in two patients and in both sputum and wound in one patient, which caused systematic infections. We found that the relapse *P. aeruginosa* isolates were mainly collected from sputum (64.5%, *n* = 40) and bronchoalveolar lavage fluid (21.0%, *n* = 13).

Most patients (77.0%, 151/196) had lung infections, followed by urinary tract infections (17.9%, *n* = 35), bloodstream infection (3.1%, *n* = 6), and wound infections (2.0%, *n* = 4). The incidence of relapse infection in patients with lung infection (39.1%, 59/151) was significantly higher than that of urinary tract infections (14.3%, 5/35, *P* = 0.0054), which indicated that the relapse *P. aeruginosa* infections usually happened in the lungs.

### Antimicrobial resistance of relapse *P. aeruginosa* isolates

**Fig 2:**
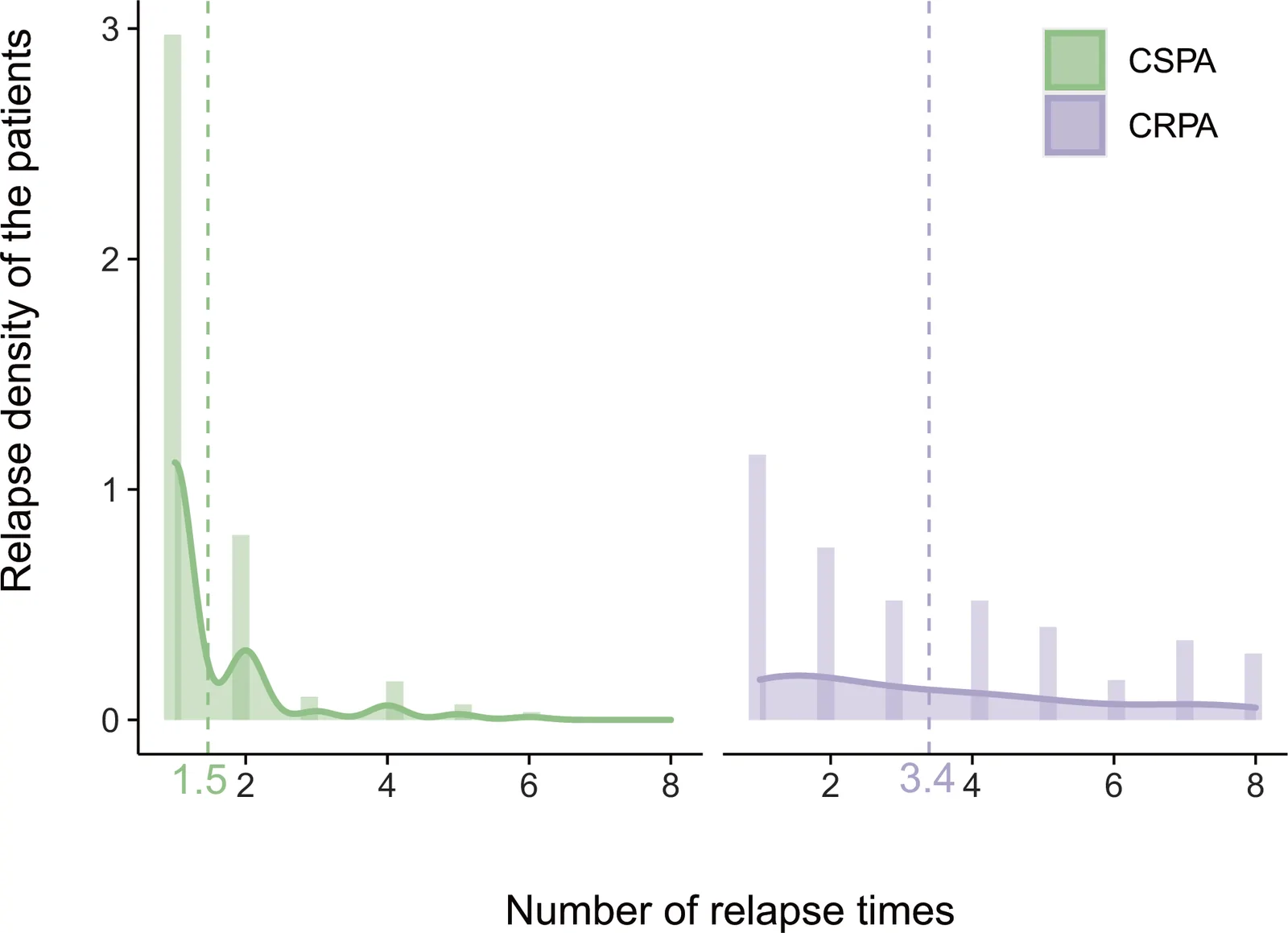
Density of relapse times for CRPA and CSPA isolates The *x*-axis represents the number of relapse times, while the *y*-axis represents the relapse density of the patients. Vertical dotted line represents the mean value of relapse times. CRPA, carbapenem-resistant *P. aeruginosa*; CSPA, carbapenem-susceptible *P. aeruginosa*.

Among 442 isolates collected from 196 patients, 41.0% (*n* = 181) of isolates were resistant to ticarcillin/clavulanate, followed by imipenem (38.5%, *n* = 170), meropenem (28.7%, *n* = 127), and levofloxacin (26.7%, *n* = 118) (Table 2). Through antimicrobial susceptibility testing (AST), we found that 41.0% (181/442) of *P. aeruginosa* isolates were resistant to carbapenems. Among 196 patients included in this study, 36.7% of patients (*n* = 72) were infected with CRPA isolates, whereas 63.3% of patients (*n* = 124) were infected with CSPA isolates. Remarkably, we observed that the incidence of relapse infection of CRPA isolates (51.4%, 37/72) was significantly higher than that of CSPA (20.2%, 25/124) isolates (*P* < 0.0001, Fig. 2), which could be attributed to the incomplete eradication since CRPA is commonly resistant to most of the antimicrobials (34).

**TABLE 2:** Antimicrobial susceptibility of 442 *Pseudomonas aeruginosa* isolates^*a*^

Antimicrobials	Antimicrobial susceptibility (*N* = 442)		
	Susceptible	Intermediate	Resistant
Ticarcillin-clavulanate	37.3% (165)	21.7% (96)	41% (181)
Cefepime	0.72 (318)	20.8% (92)	7.2% (32)
Ceftazidime	74.4% (329)	5.2% (23)	20.4% (90)
Amikacin	93.2% (412)	1.8% (8)	5% (22)
Tobramycin	91.4% (404)	1.1% (5)	7.5% (33)
Meropenem	61.8% (273)	9.5% (42)	28.7% (127)
Imipenem	0.61 (270)	0.5% (2)	38.5% (170)
Ciprofloxacin	0.79 (349)	10.6% (47)	10.4% (46)
Levofloxacin	63.8% (282)	9.5% (42)	26.7% (118)
Colistin	95.9% (424)	–	4.1% (18)

^*a*^ The numbers in the table refer to the percentages of isolates showing susceptibility, intermediate susceptibility, and resistance to each antimicrobial. The numbers in parentheses indicate the number of isolates. –, not applicable.

During the different relapse episodes, the antimicrobial resistance spectrum of *P. aeruginosa* isolates changed in 17 patients (27.0%, *N* = 63). Decreased susceptibility of antimicrobials was observed in 15 patients, including imipenem (*n* = 13), meropenem (*n* = 13), ticarcillin-clavulanate (*n* = 8), cefoperazone/sulbactam (*n* = 5), ceftazidime (*n* = 5), and amikacin (*n* = 2), whereas increased susceptibility of ciprofloxacin and levofloxacin was observed in one patient and imipenem and meropenem in another one (Table S2). These phenotypical changes could be ascribed to antimicrobial therapy during relapse episodes, reflecting a fast adaptation of *P. aeruginosa* facing antimicrobial selective pressures.

### Virulence-associated genes, antimicrobial resistance genes, and plasmid of relapse *P. aeruginosa* isolates

Among 156 isolates from patients with relapse *P. aeruginosa* infection, a total of 60 kinds of virulence-associated genes and 2 kinds of plasmid replicons were identified against VFDB and PlasmidFinder databases (Tables S4 and S5). The average number of virulence-associated genes for each isolate is 48.4 (standard deviation [SD]: ±3.7). A total of 45 isolates harbored IncFIA plasmid, and 12 isolates harbored IncQ plasmid. No VFs and plasmid replicons were lost or acquired among different time points of infections in all patients.

A total of 25 ARGs were detected among relapse *P. aeruginosa* isolates (Table S3). The average number of ARGs for each isolate is 5.7 (SD: ±1.1). We found that 41 patients had the same ARGs among different time points of infections. Notably, the *P. aeruginosa* isolates of nine patients lost ARGs at the later stages of infections, such as *aph(3')-IIa* (*n* = 5) and *aac (3)-IV* (*n* = 5), while *P. aeruginosa* isolates of 10 patients acquired new ARGs at a later stage, such as *catA1* (*n* = 7) and *aac (3)-IV* (*n* = 5). Acquisition and loss of the entire genes are important for evolution and adaptation during infection, which is commonly connected with horizontal transfer of mobile genes and transposons that can rapidly introduce large genomic and phenotypic changes, conferring antimicrobial resistance, virulence, or fitness advantage (12, 13, 35).

### Genomic characterizations of relapse *P. aeruginosa* isolates

These relapse *P. aeruginosa* isolates were assigned into 50 distinct STs, of which 8 new STs were identified among 16 isolates (Fig. 4; Table S6). Notably, our results showed that four patients were infected with ST357 isolates and three patients with ST1182 isolates, while 40 STs were detected in only one patient and 8 STs in two patients, which indicated that the relapse infections were not caused by certain lineages of *P. aeruginosa*.

**Fig 3:**
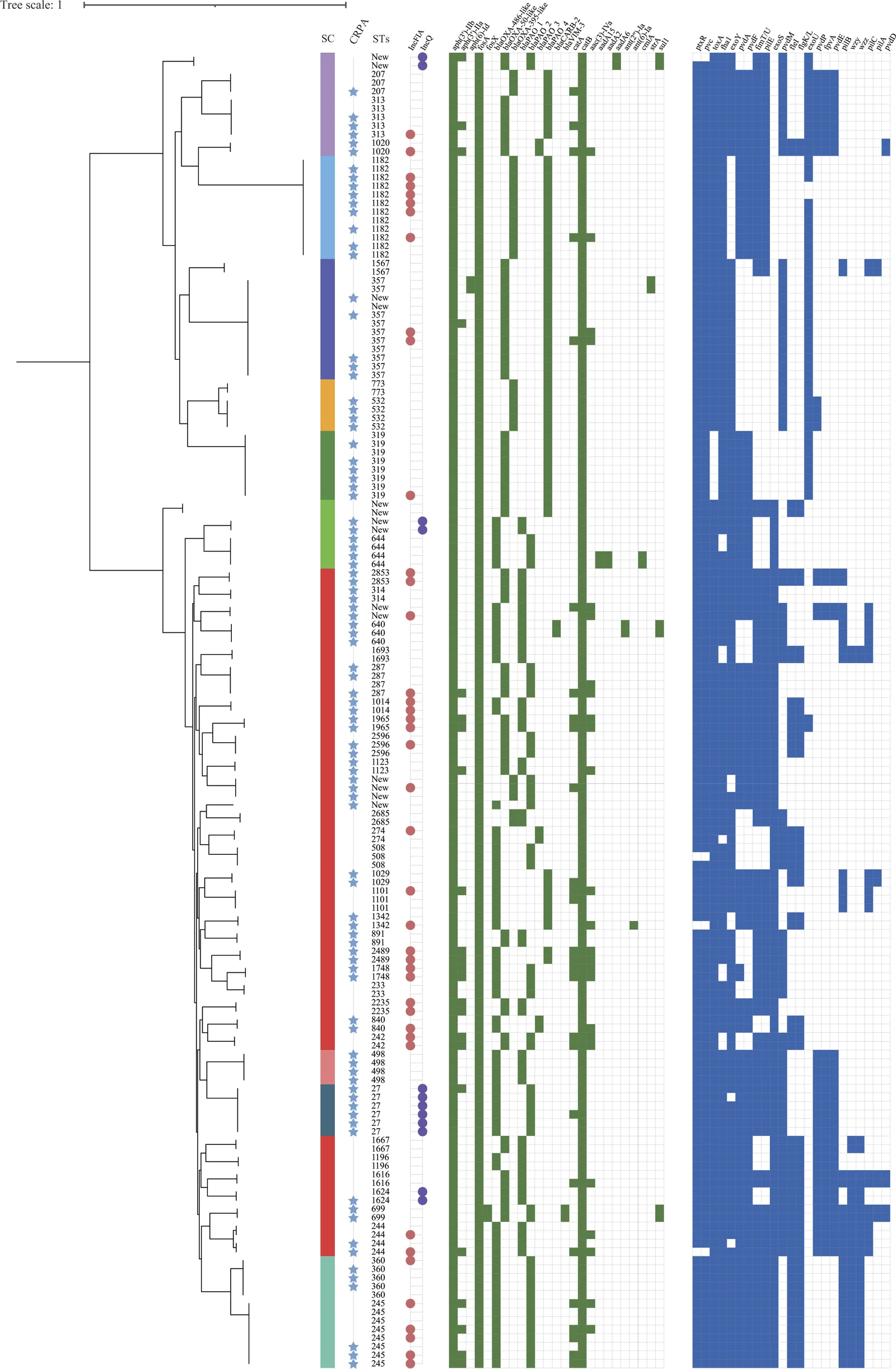
Phylogenetic tree and genomic characteristics of *P. aeruginosa* isolates from relapse infections. The ML phylogenetic tree was constructed by cgSNPs of 156 *P*. *aeruginosa* isolates from 62 patients with relapse *P. aeruginosa* infections. The sequence clusters (SCs), CRPA, MLST, and plasmid replicon were labeled by a color strip, star, text, and color dot. In the heatmap, green blocks denote the presence of an ARG, while blue blocks denote the presence of a virulence factor.

**Fig 4:**
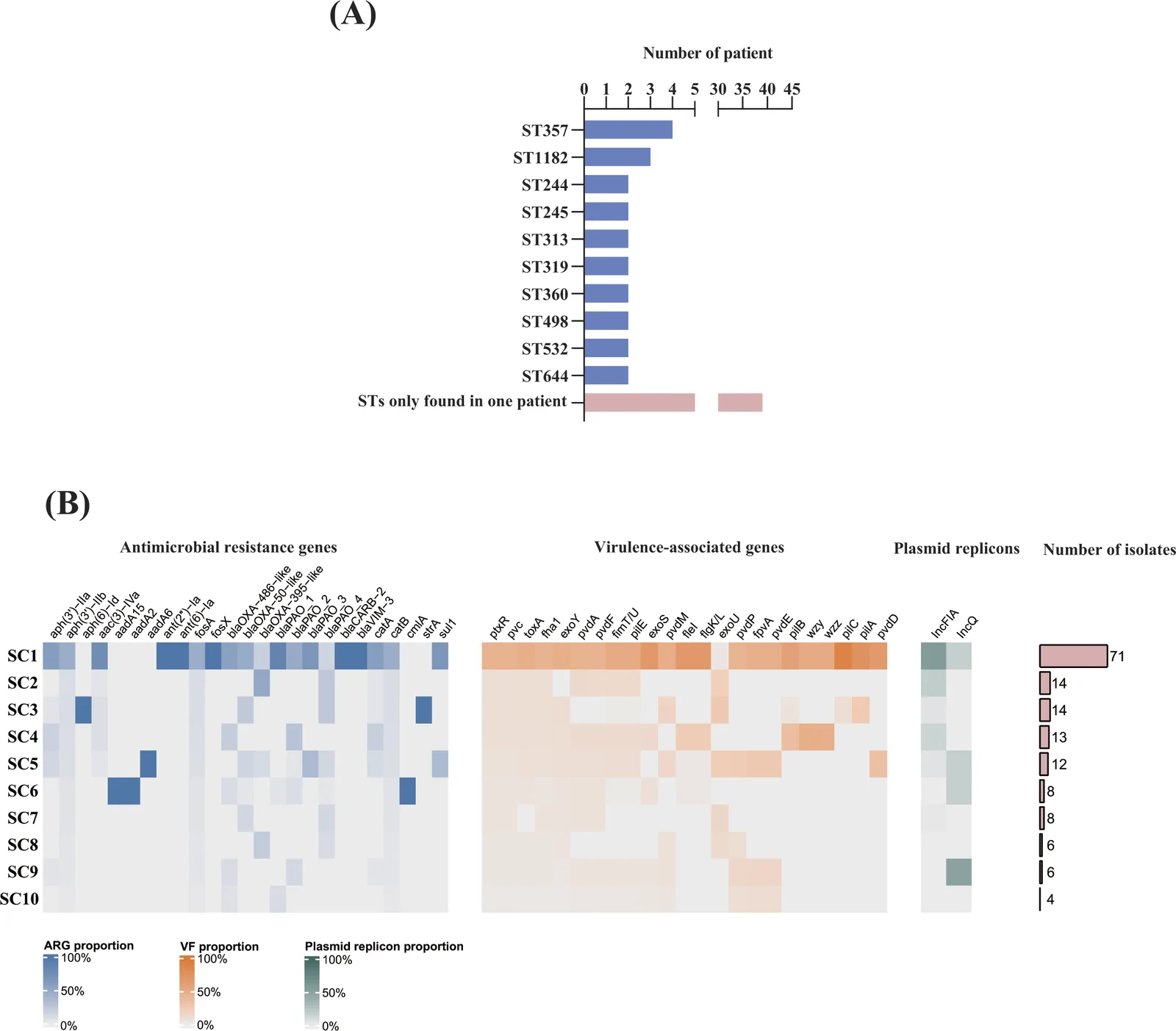
MLST, ARGs, virulence-associated genes, and plasmid replicons for each sequence cluster based on phylogeny. (**A**) The distribution of MLST. The pink bar represents the number of patients of which ST is only found in one patient. (**B**) The proportion heatmap of ARGs, VFs, and plasmid replicons for each sequence cluster. The proportions of ARGs, virulence-associated genes, and plasmid replicons were represented by blue, orange, and green heatmaps, respectively.

Pan-genome analysis of 156 relapse *P. aeruginosa* isolates from 62 patients identified 4,785 core genes representing a 4.5-Mb alignment in ≥99% of genomes. hierBAPS analysis of the relapse *P. aeruginosa* population structure based on 59,631 cgSNPs identified 10 sequence clusters (SCs), of which SC7 was polyphyletic in the ML phylogeny and bifurcated as two distinct monophyletic clades (Fig. 3). SC7 was the most prevalent cluster containing 46.8% (73/156) of isolates (Fig. 4). We observed that the relapse *P. aeruginosa* isolates were distributed into different branches, and the distribution of ARGs, VFs, and plasmid replicons was scattered, indicating no lineage specificity in relapse *P. aeruginosa* isolates (Fig. 3).

### Pairwise genetic distance and evolution of relapse *P. aeruginosa* isolates

**Fig 5:**
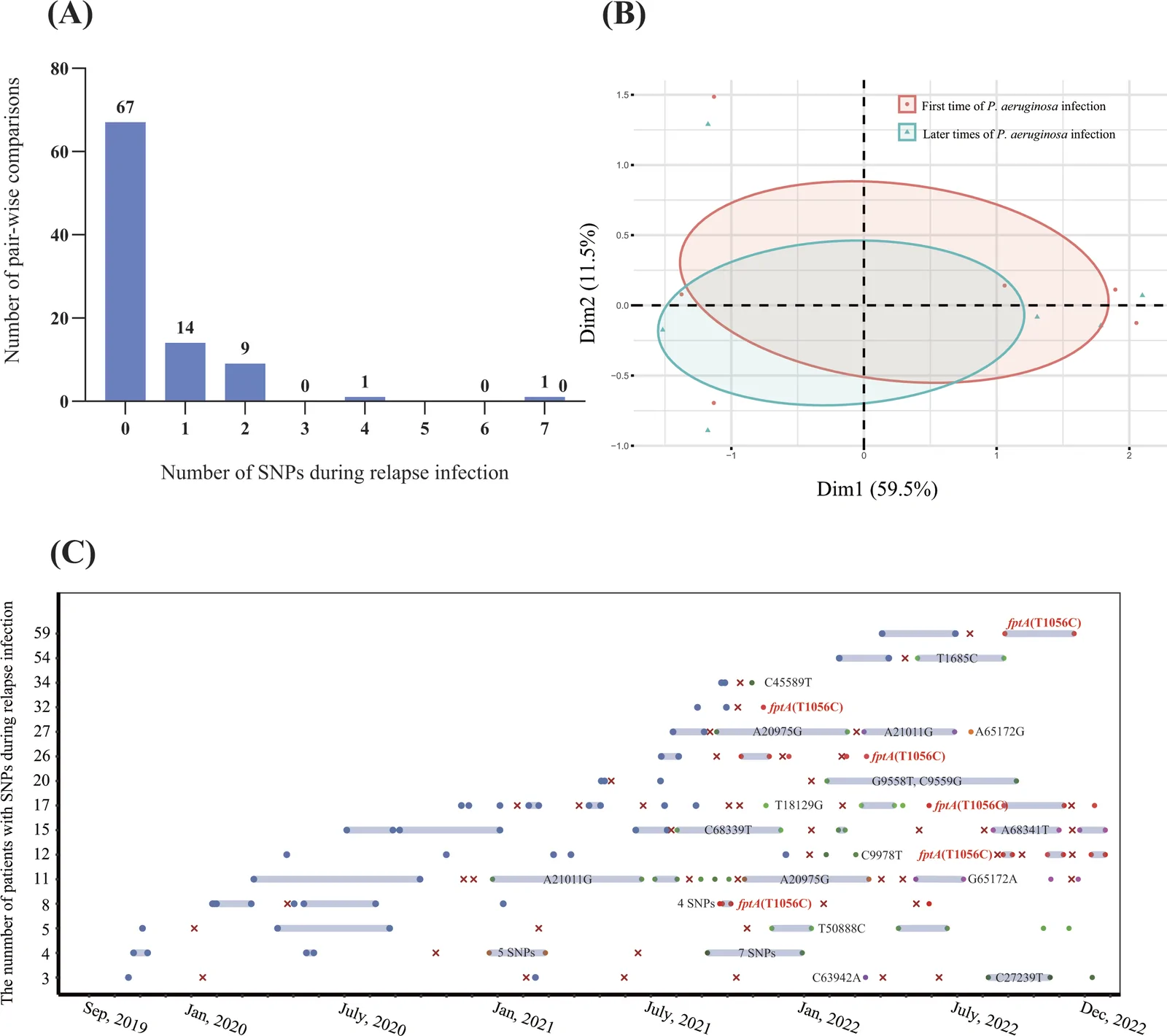
Dynamic of SNPs in *P. aeruginosa* genome during the episodes of relapse infection (**A**) Number of SNPs in *P. aeruginosa* core genome among pair-wise comparisons during relapse infection of 62 patients. (**B**) PCA analysis of cgSNPs among first time and later times of relapse *P. aeruginosa* isolates. (**C**) Timelines for each patient with SNPs during the episodes of relapse infections. Data are shown for isolates of 15 patients from 1 September 2019, to 31 December 2022. The blue dots represent the positive cultures with *P. aeruginosa* isolates. The blue dots represent the genotype of *P. aeruginosa* isolate for each patient. The red dots labeled with red text represent SNP found in the fptA gene (T1056C). The green, purple, and orange dots represent the SNPs found in other SNP sites.

Previous studies demonstrated that micro-evolution and adaptation could happen in *P. aeruginosa* isolate under the selective pressure of antimicrobials and host immune systems, which could promote virulence and antimicrobial resistance of *P. aeruginosa* isolates (5, 12 – 15) . Therefore, we performed a genomic comparative analysis to reveal the evolution of relapse *P. aeruginosa* isolates. The results of pairwise SNP distance of *P. aeruginosa* isolates for each patient showed that SNPs of the isolates from 15 patients occurred during early and subsequent stages of relapse infections (ranging from one to seven SNPs), whereas 47 patients occurred with no SNP among different time points (Fig. 5A). These SNPs were located on 25 sites of *P. aeruginosa* chromosome, and 24 of them are detected in only one patient.

Principal component analysis (PCA) showed that *P. aeruginosa* isolates among the first episode and later episodes were divided into two parts, which may reveal that there is a convergent evolution during the episodes of relapse infections (Fig. 5B). Remarkably, we found an SNP, which causes a non-synonymous mutation in the *fptA* gene (T1056C, M252T), was detected in relapse *P. aeruginosa* of six patients (Fig. 5C). FptA, a TonB-dependent transporter, promotes the utilization of iron that permits the high-affinity binding and transport of Fe(III)-pyochelin complex across the outer membrane, which is associated with the virulence of *P. aeruginosa* (36, 37). The mutation of the *fptA* gene has also been detected in patients with long-term *P. aeruginosa* infections, which indicated that convergent microevolution occurs in relapse *P. aeruginosa* isolates (38).

## DISCUSSION

Relapse *P. aeruginosa* infection is responsible for repeated admissions and adverse outcomes of patients. In European and American countries, relapse *P. aeruginosa* infection commonly happened in CF patients. However, the prevalence and characteristics of relapse *P. aeruginosa* infections in China remain unknown since CF is a rare disease in China (16). In this study, we performed a 3-year retrospective study to investigate the epidemiological and genomic characteristics of *P. aeruginosa* isolates causing relapse infections in a tertiary hospital in China.

Whole-genome sequencing is a precise method for recognition among relapse infection, persistent infection, and reinfection of a certain pathogen (6 – 8). Our results showed a high accuracy (95.4%, 62/65) in identifying relapse infections by screening clinical records using the standards in this study. However, the distinguishment between relapse and persistent infections relied on both the genotyping by WGS and clinical symptoms. In many previous studies, relapse and persistent infections were collectively analyzed since the clinical symptoms during the treatment periods were ignored, which underestimated the influences of entirely different dynamics of pathogen virulence and the host’s immune status (10, 11, 14, 38 – 40).

Our results revealed that the incidence of relapse *P. aeruginosa* infection in this study (31.6%, 62/196) is high but relatively lower than the results from European and American countries, which could be attributable to the reason that CF patients were not found in our cohort, which is an important risk factor for colonization and infection of *P. aeruginosa* (13, 41). Relapse *P. aeruginosa* infections are commonly detected in the lungs (91.9%, 57/62), indicating that the specific host lung environment favors *P. aeruginosa* colonization and persistence. Notably, 30.8% (20/65) of the patients were diagnosed with chronic lung diseases, but no direct indicator was found. Besides, we found that most of the patients are all elderly and with prolonged hospitalization which are significant risk factors for *P. aeruginosa* colonization (42). Therefore, we propose that decolonization could be an efficient therapy for the treatment and prophylaxis of relapse *P. aeruginosa* infection.

We revealed that the incidence of relapse in CRPA isolates (51.4%, 37/72) is significantly higher than that in CSPA isolates (20.2%, 25/124, *P* < 0.0001), which could be attributed to the reason that CRPA is commonly resistant to most of the antimicrobials and hard to eradicate completely (34). Decreased susceptibility of several antimicrobials was observed in 27% (7/63) of patients in the late stage of relapse, including carbapenems, β-lactams, and aminoglycosides. Besides, we found that two aminoglycoside resistance genes (*aph(3')-IIa* and *aac(3)-IV*) underwent dynamic events of loss or acquisition in the late stage of relapse infection. As previously confirmed, the acquisition of ARGs could confer resistance, whereas the loss of ARGs could confer a fitness advantage, which is important for evolution and adaptation during infection (13, 35). However, only one-half of patients (4/10) have received aminoglycoside treatment, which may imply that phenotypical and genotypical dynamics of antimicrobial resistance reflect fast adaptation and genomic evolution not just in antimicrobial resistance but also in changing fitness, resulting in incomplete eradication of *P. aeruginosa* isolates or increased bacterial fitness by lost genes with a burden.

The identification of pathoadaptive genes, in which mutations accumulate with higher frequencies than expected statistically during relapse infection, gave hope to finding genetic markers that might predict the potential to establish chronic infection in the clinic. In our study, the results showed that relapse infections are not caused by specific lineages of *P. aeruginosa* isolates since these isolates are assigned to distinct STs and sporadically distributed in the phylogenetic tree. Therefore, we proposed that the relapse infection could impute to non-lineage-associated factors, such as genetic evolutions of horizontal gene transfer, mutation, or relapse-associated functional virulence-associated genes. To test our hypothesis, we additionally analyzed the genetic evolution at different times of relapse *P. aeruginosa* isolates. We found several mutations of cgSNPs in the later time of relapse infections, reflecting a dramatic genetic evolution during relapse infections. Notably, we found a convergent evolution of a non-synonymous mutation in the *fptA* gene (T1056C, M252T). FptA is a TonB-dependent transporter that permits the high-affinity binding and transport of Fe(III)-pyochelin complex, driving the import of the bacteriocin across the outer membrane and promoting the virulence of *P. aeruginosa*, especially in the pulmonary and urinary tract infections (43, 44). Consistently, another study has found that the *fptA* gene was hypermutated in *P. aeruginosa* isolates collected from prolonged ICU inpatients; even the epidemiological and genomic information of these isolates were entirely different from the isolates in this study (38). Since the host lung microbiome is able to guard against colonization and infection of pathogens by competing for the nutrient metal iron (40), the *P. aeruginosa* is warranted to increase its metal iron acquisition function to overcome the iron limitation which could be a selective pressure facilitating the microevolution of *fptA* gene, thus in favor of the fitness and adaptation of *P. aeruginosa* isolates in relapse and prolonged infections. Previous studies have indicated that FptA confers increased susceptibility to siderophore-drug conjugates and is considered a novel target for treating multidrug-resistant *P. aeruginosa* (37). According to the above evidence, we speculate that FptA could be a considerable target for diagnostics, decolonization, and treatment of relapse and prolonged infections by *P. aeruginosa* isolates.

Our study has several limitations. First, we included the patients with *P. aeruginosa* infections in a single clinical center, and our epidemiological findings may not be generalizable. We realize that more wide-ranging studies involving the surveillance and genomic investigations are needed to clarify the prevalence and genomic characterizations of relapse *P. aeruginosa* infection since CF is a rare disease in China. Secondly, we are not able to trace the clinical records of patients with relapse infections who were hospitalized in other medical centers, which could underestimate the incidence of relapse *P. aeruginosa* infections. Thirdly, we were not able to consider the influence of selective pressure from the host immune environment on the convergent evolution of relapse *P. aeruginosa* isolates. Above two limitations were restricted by the retrospective study design. A prospective cohort study and the collection of comprehensive clinical samples would supply strong evidence to address these gaps. Finally, we were not able to assess the causality between our genomic findings and relapse infection based on an epidemiological study. Further metatranscriptomics and experimental evidence would be helpful to address this noteworthy question and is part of our future work.

Despite these limitations, our data provide a comprehensive understanding of the high prevalence and genomic dynamics of relapse *P. aeruginosa* isolates across different stages. Integrated utilization of WGS and medical records provides opportunities for improved diagnostics of relapse infections. Relapse *P. aeruginosa* infections were not caused by a certain lineage of isolates but could be attributable to the convergent evolution of the siderophore-encoding gene. Continued surveillance of the genomic dynamics of relapse *P. aeruginosa* infection will generate further knowledge for optimizing the treatment and prevention of relapse infection in the future, and our approach could potentially be used as a template for monitoring and diagnosing relapse infection of such prolonged infection by pathogens more widely.

## Data Availability

The genome assemblies of *P. aeruginosa* reported in this study have been deposited in the NCBI GenBank genomic DNA database under BioProject accession number PRJNA974176.

## SUPPLEMENTAL MATERIAL

Supplemental legends spectrum.05312-22-s0001.docx Legends for Tables S1 to S6.

Supplemental tables spectrum.05312-22-s0002.xlsx Tables S1 to S6.

ASM does not own the copyrights to Supplemental Material that may be linked to, or accessed through, an article. The authors have granted ASM a non-exclusive, world-wide license to publish the Supplemental Material files. Please contact the corresponding author directly for reuse.
