# Supplementary material for: Molecular epidemiology and genomic dynamics of Pseudomonas aeruginosa isolates causing relapse infections
Source: Microbiol Spectr. 2023 Sep 28;11(5):e05312-22. doi: 10.1128/spectrum.05312-22 (PMC10581123; doi:10.1128/spectrum.05312-22)
Supplement: Supplemental legends — Legends for Tables S1 to S6. [file spectrum.05312-22-s0001.docx]

**Supplement table legends**

Supplement table 1. Clinical information of 196 patients included in this study.

Supplement table 2. Antimicrobial resistance patterns and MICs of 442 *P. aeruginosa* isolates.

Supplement table 3. ARGs of 156 *P. aeruginosa* isolates from relapse infections.

Supplement table 4. Virulence-associated genes of 156 *P. aeruginosa* isolates from relapse infections.

Supplement table 5. Plasmid replicons of 156 *P. aeruginosa* isolates from relapse infections.

Supplement table 6. MLST and sequence clusters of 156 *P. aeruginosa* isolates from relapse infections.
